# The Expression of p-STAT3 in Stage IV Melanoma: Risk of CNS Metastasis and Survival

**DOI:** 10.18632/oncotarget.475

**Published:** 2012-04-06

**Authors:** Ian Lee, Patricia S. Fox, Sherise D. Ferguson, Roland Bassett, Ling-Yuan Kong, Christopher W. Schacherer, Jeffrey E. Gershenwald, Elizabeth A. Grimm, Gregory N. Fuller, Amy B. Heimberger

**Affiliations:** ^1^ Department of Neurosurgery, The University of Texas MD Anderson Cancer Center, Houston, Texas; ^2^ Department of Biostatistics,The University of Texas MD Anderson Cancer Center, Houston, Texas; ^3^ Department of Surgical Oncology, The University of Texas MD Anderson Cancer Center, Houston, Texas; ^4^ Department of Melanoma Medical Oncology,The University of Texas MD Anderson Cancer Center, Houston, Texas; ^5^ Department of Pathology, The University of Texas MD Anderson Cancer Center, Houston, Texas

**Keywords:** melanoma, p-STAT3, CNS metastasis, prognosis

## Abstract

**Purpose:**

The signal transducer and activator of transcription 3 (STAT3) is a key molecular hub of tumorigenesis and immune suppression. The expression of phosphorylated STAT3 (p-STAT3) has been shown to be higher in melanoma metastasis to the central nervous system (CNS) relative to distant metastasis in the rest of the body (systemic). We sought to determine whether the increased expression of p-STAT3 in non-CNS systemic melanoma metastasis is associated with an increased risk of developing CNS metastasis and is a negative prognostic factor for overall survival time.

**Methods:**

We retrospectively identified 299 patients with stage IV melanoma. In a tissue microarray of systemic non-CNS metastasis specimens from these patients, we used immunohistochemical analysis to measure the percentage of cells with p-STAT3 expression and Kaplan–Meier survival estimates to analyze the association of p-STAT3 expression with median survival time, time to first CNS metastasis, and development of CNS metastasis.

**Results:**

Lung metastases exhibited the highest level of p-STAT3 expression while spleen lesions had the lowest. The p-STAT3 expression was not associated with an increased risk of developing CNS metastasis or time to CNS metastasis. However, p-STAT3 expression was a negative prognostic factor for overall survival time in patients that did not develop CNS metastasis.

**Conclusions:**

Stage IV melanoma patients without CNS metastasis treated with p-STAT3 inhibitors in efficacy studies should be stratified based on tumor expression of p-STAT3; however since p-STAT3 expression is not associated with the risk of CNS disease, increased MRI surveillance of the brain is not likely necessary.

## INTRODUCTION

Although very early stage melanoma has an excellent prognosis, with 10-year survival rates as high as 95%, once distant metastases develop, survival drops to 8-20% at 10 years [1]. Unfortunately, melanoma has a high propensity to metastasize to the central nervous system (CNS). It is the third most common cause of brain metastases, after lung cancer and breast cancer [2]. In fact, over their lifetime, CNS involvement is reported in 46% of melanoma patients [3, 4]. The incidence of CNS metastasis in melanoma patients ranges from 50% to 70% in autopsy series [5, 6]. Once melanoma has metastasized to the brain, the prognosis is dismal despite multi-modality therapy. CNS recurrence is often the first site of progression seen in up to 25% of responsive patients [7] and the median survival time in melanoma is approximately 5 months in large published series [8]. As many as 70% of patients who develop CNS metastases have multiple symptomatic lesions and hence are often ineligible for clinical trials [9]. As such, the determination of the factors leading to the development of distant melanoma metastases, especially CNS metastases, and the elucidation of potential agents to block the effects of these factors is an area of intense investigation.

A key transcription factor, STAT3, has been shown to drive the fundamental components of tumorigenesis and metastasis by preventing apoptosis (by increasing expression of survivin, BCL-XL, and MCL1) and enhancing proliferation (by increasing expression of c-Myc and cyclin D1/D2) [10], angiogenesis (by increasing expression of VEGF and hypoxia-inducible factor-1α), and metastasis (by increasing expression of MMP-2 and MMP-9) [11, 12]. Growth factors and cytokines, including interleukin (IL)-6, can activate STAT3 by phosphorylating the tyrosine residue in the STAT3 transactivation domain [13]. The p-STAT3 then translocates into the nucleus and induces the expression of a variety of target genes. STAT3 is constitutively overexpressed in many different cancers; in melanoma, 81% of CNS metastases express activated STAT3 [14]. Preclinical studies using decoy antisense STAT3 oligonucleotides, dominant-negative vectors, and small-molecule inhibitors have provided convincing evidence that STAT3 is highly relevant to the growth and survival of many tumor types [15-21], *in vitro* and *in vivo*. Likewise, over expression of a constitutively active p-STAT3 mutant has been found to induce tumor growth in murine and *in vitro* studies [22, 23]. Furthermore, STAT3 has been shown to be a key regulator of tumor-mediated immune suppression [24, 25]. Xie et al. demonstrated that highly metastatic melanoma cell lines have higher levels of p-STAT3 than do poorly metastatic ones [26]. In addition, by blocking activated p-STAT3 in highly metastatic melanoma cells, the invasiveness and tumor growth were significantly suppressed. Consequently, metastases were able to be prevented in nude mice implicating p-STAT3 in the development of distant metastasis [26]. Finally, p-STAT3 levels have been found to be higher in brain metastases than in cutaneous primary melanomas [14], further highlighting the possible role of p-STAT3 in the development of metastases, especially to the CNS. Thus, *we hypothesized that higher systemic (non-CNS distant metastasis) tumor levels of p-STAT3 would predispose patients to the development of CNS metastasis and would be a negative prognostic factor for survival.*

## Materials and Methods

### Ethics Statement

This study was conducted according to LAB09-0463 approved by the Institutional Review Board of The University of Texas MD Anderson Cancer Center in which 299 patients with stage IV melanoma were identified from the MD Anderson Cancer Center Melanoma Informatics, Tissue Resource, and Pathology Core (MelCore) database. Surviving patients provided their written consent for their clinical information to be stored in the MelCore database and the use of their tissue under LAB03-320 and LAB00-063. Minors were not involved in this study.

### Melanoma Tissue Microarray (TMA)

Patients included those with systemic, non-CNS metastasis samples accessioned at MDACC between 1992 and 2010. Data entry was finalized on October 27, 2011. The composition of the TMA consisted of resected metastasis from the lung (n = 155), intestine/colon (n = 79), liver (n = 18), adrenal gland (n = 13), spleen (n = 12), gall bladder (n = 5) and other non-CNS sites (n = 17). Of these patients, 148 had or developed radiographic confirmed CNS metastases. For TMA construction, two 1mm cores where obtained per tumor sample. The rationale for using a TMA was to facilitate analysis of the largest number of cases possible. The study neuropathologist (GNF) gathered the tissue sections and confirmed the tumor pathology by using the archived paraffin-blocked tissue sections. The time from resection to fixation was less than 20 minutes in all cases in accordance with the CLIA (Clinical Laboratory Improvement Amendments) standard.

### Immunohistochemical analysis of p-STAT3 expression

The method for detecting and determining p-STAT3 expression has been previously described [27]. Briefly, formalin-fixed, paraffin-embedded 5μm sections of the melanoma TMA were first deparaffinized in xylene and rehydrated in ethanol. The endogenous peroxidase activity was blocked with 0.3% hydrogen peroxide/methanol for 10 minutes at room temperature before antigen retrieval was begun. The ThermoScientific PT Module (Thermo Fisher Scientific, Fremont, CA) with citrate buffer (pH 6.0) was then used for antigen retrieval. After blocking with a serum-free protein block solution (DAKO, Carpinteria, CA), diluted anti-p-STAT3 (tyrosine^705^) antibody (1:50; Cell Signaling Technology, Danvers, MA) was added to the TMA and the specimens were incubated overnight in a humidified box at 4°C. The slides were then stained with a biotin-labeled secondary antibody (biotinylated link universal solution; DAKO) for 60 minutes at room temperature. Finally, streptavidin-horseradish peroxidase (DAKO) was added and incubated with the slides for 30 minutes at room temperature. Diaminobenzidine (DAKO) was used as the chromogen, and color development was halted by dipping the slides in distilled water. The nuclei were then counterstained with hematoxylin. For the negative control, an isotype antibody was used (Santa Cruz Bio-technology, Santa Cruz, CA).

### Quantification of p-STAT3 expression

**Figure 1:**
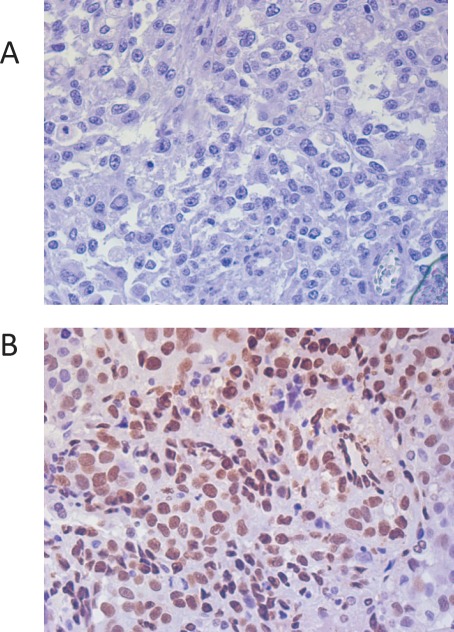
Immunohistochemical staining of melanoma tissue sections demonstrating p-STAT3 staining confined to the nucleus Representative negative (A) and positive (B) specimens are shown (400x magnification).

Independent observers (IL, SF) quantitatively evaluated p-STAT3 expression by analyzing the cores using high-power fields (maximum: x40 objective and x10 eyepiece) of each specimen (Fig. 1). These evaluations were validated by the study pathologist (GNF). The observers examined each sample in duplicate cores from different areas of the same tumor in a blinded fashion. Up to six observations were made of the metastatic tissue sample from each patient. For each observation, the total number of cells and the number of cells with positive p-STAT3 nuclear staining were counted. The percentage of p-STAT3 positive cells was then calculated for each observation. The average percentage of cells with positive p-STAT3 nuclear staining was calculated from all observations for each patient's sample. For most patients, this was the average of six sets of counts. Hereafter, this average percentage of p-STAT3-positive cells is referred to as the p-STAT3 expression level. Observations in which no cells could be counted were excluded. We minimized potential mismatching of the data by staining an intact TMA with hematoxylin and eosin and identifying the correct location of each tissue core by visually matching the tumors on the basis of their unique histologic elements.

### Statistical analysis

The Wilcoxon rank-sum test was used to compare the distribution of p-STAT3 expression between patients with and without CNS metastasis, and the Kruskal-Wallis test was used to compare the distribution of p-STAT3 expression among tissue types. The tissue type denotes the organ from which the melanoma metastasis specimen was taken. Median survival was computed from the date of stage IV diagnosis to the date of the patient's last known vital status. The time to first CNS metastasis was computed from the date of stage IV diagnosis to the date of first CNS metastasis. Patients with preceding CNS metastasis or CNS metastasis at the time stage IV diagnosis were excluded (n = 21). Patients that did not experience a CNS metastasis were censored by using the date of the patient's last brain imaging assessment or the last date the patient was seen by a physician at MD Anderson Cancer Center. Kaplan-Meier survival curves were used to estimate survival [28], and the log-rank test was used to assess differences between groups of patients with different levels of p-STAT3 expression [29]. Cox proportional hazards regression was used to analyze the association between p-STAT3 expression and survival. Logistic regression was used to analyze the association between p-STAT3 expression and the development of CNS metastasis. The p-STAT3 was considered first as a continuous variable, and secondary analyses were performed separately which categorized patients as having p-STAT3 expression < 1% vs. ≥ 1%. We decided on this dichotomization based on its possible clinical relevance after analyzing absolutely none versus any p-STAT3 expression as well as comparing percentiles that represented the highest and lowest levels of expression. Additionally, in the literature [30], it is acceptable to use a minimum percentage of expression as evidence of expression rather than dichotomizing based on zero. Because the distribution of p-STAT3 was positively skewed (median = 8.99%, range 0 - 74%), transformed expression values were analyzed but did not yield significantly different results and are not reported. A *p*-value of less than 0.05 was considered significant. All statistical analysis were performed using SAS software version 9.2 for Windows (SAS Institute, Cary, NC).

## RESULTS

### Study population

**Figure 2:**
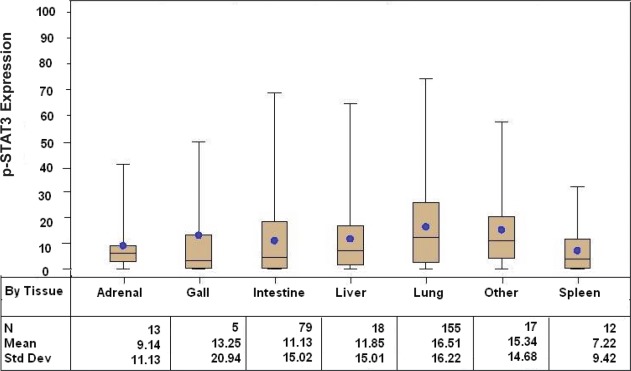
Box and whisker plots stratified by systemic organ metastasis site demonstrating p-STAT3 expression, as determined by immunohistochemical staining, among patients with stage IV melanoma (*p* = 0.0155 across all tissue types)

Tissue sections from 299 patients with distant metastatic melanoma were included in the TMA of which 151 (51%) did not have CNS metastasis at the time of the last follow-up. The median age of the patients at initial diagnosis was 50 years (range: 12 – 80 years) and 56 years at the time of the stage IV diagnosis (range: 13 – 85 years). The majority were caucasian (95%) and male (71%). Figure 2 summarizes the overall composition of the melanoma TMA, which consisted of resected metastasis from the lung (n = 155), intestine/colon (n = 79), liver (n = 1 8), adrenal gland (n = 13), spleen (n = 12), gall bladder (n = 5) and other (n = 17). The most common site of melanoma metastasis was the lung, consistent with previous reports [31]. The distribution of p-STAT3 expression was significantly different across all tissue types (*p* = 0.0155), with the highest expression seen in the lung (mean 16.5%, median 12.3%).

### The expression of p-STAT3 does not impact overall survival in stage IV melanoma patients

**Figure 3:**
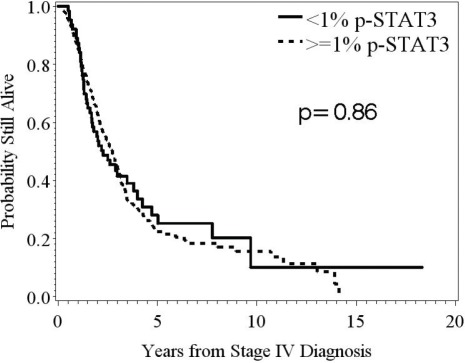
Kaplan-Meier survival estimates stratified by p-STAT3, expression determined from immunohistochemical staining, in patients with stage IV melanoma In melanoma patients stratified based by the amount of p-STAT3 expression <1% versus ≥1%, there was no significant difference in median survival time (*p* = 0.86; n = 63, 236, respectively).

Cox proportional hazard regression was used to determine whether p-STAT3 expression was a significant predictor of survival. For all stage IV melanoma patients, the overall median survival was 2.7 years. Intratumoral, nuclear p-STAT3 expression was not an independent univariate predictor of overall survival in stage IV melanoma patients (HR = 1.008; 95%CI: 0.998-1.015; n deaths = 222; *p* = 0.13) (Fig. 3).

Because we had observed a statistically significant difference between p-STAT3 expression levels in melanoma metastasis of different tissue types, we conducted a sub-analysis of survival by tissue type using the two tissue types with the greatest number of samples. Patients with metastasis originating from the intestine and lung were selected to represent the higher and lower ends of the p-STAT3 spectrum, respectively. We found no significant differences in survival in patients with metastasis to either of these tissue types based on the amount of p-STAT3 expression, validating our finding that p-STAT3 expression is not a prognostic marker in patients with stage IV melanoma.

### The expression of p-STAT3 is not predictive of development or time to CNS metastasis

Among the systemic melanoma metastasis of stage IV melanoma patients without CNS metastasis (n=151), p-STAT3 expression was 14.7% (SD = 14.6); whereas it was 13.3% (SD = 16.5) in the systemic melanoma metastasis of those patients with CNS metastasis (n=148). Although there was some evidence of a difference in the distribution of p-STAT3 by CNS metastasis status among these stage IV melanoma patients (*p* = 0.05); this small difference may not be clinically meaningful and may be attributable to the fact that there were more lung samples, which have higher p-STAT3 expression, in the group without CNS metastasis. Univariate logistic regression analysis revealed that p-STAT3 expression (when defined as a continuous variable) in systemic melanoma metastasis was not predictive for the development of CNS metastasis (HR: 0.994; 95% Wald CI: 0.980 – 1.009; n metastases = 148; *p* = 0.44).

**Figure 4:**
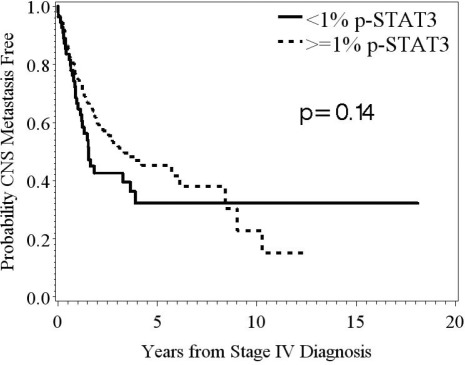
Kaplan-Meier survival estimates of the overall probability of developing CNS metastasis from the time of the stage IV diagnosis In patients stratified based on the amount of p-STAT3 expression, <1% versus ≥1%, in their systemic metastasis, there was no significant difference in time to development of CNS metastasis (*p* = 0.14; n = 55, 223, respectively).

The median time to the development of CNS metastasis was 3.0 years from the time of the stage IV diagnosis. We found no significant differences in time to CNS metastasis based on the amount of p-STAT3 expression in the systemic melanoma metastasis (Fig. 4).

### The expression of p-STAT3 in stage IV melanoma patients without CNS metastasis impacts survival

**Figure 5:**
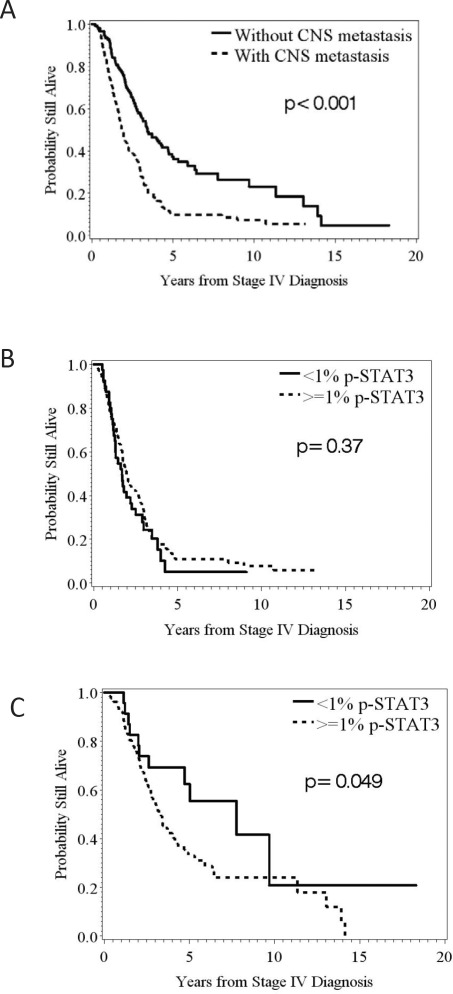
Kaplan-Meier survival estimates of the overall probability of survival from the time of the stage IV diagnosis (A) The median survival was 1.9 years in those patients that developed CNS metastasis compared to 3.4 years in those patients that did not. (B) In melanoma patients that developed CNS metastasis stratified by the amount of p-STAT3 expression, <1% versus ≥1%, in their systemic metastasis, no significant difference was found in survival (*p* = 0.37; n = 40, 108, respectively). (C) In melanoma patients that did not develop CNS metastasis, significant difference were found in survival based on the amount of p-STAT3 expression, <1% versus ≥1%, in their systemic metastasis (*p* = 0.049; n = 23, 128, respectively).

Since the presence of CNS metastasis has been previously demonstrated to be a negative prognostic factor for survival in stage IV melanoma patients, we evaluated this parameter within our dataset and validated this previously identified prognosticator (Fig. 5A; log-rank *p* < 0.001). The overall median survival was 1.9 years in those patients with CNS metastasis compared to 3.4 years in those patients who had not developed CNS metastasis. We further analyzed the association between p-STAT3 and overall survival separately by CNS metastasis status. No prognostic impact was found within the subset of patients that had developed CNS metastasis based on the systemic p-STAT3 expression (Fig. 5B). However, within the subset of patients without CNS metastasis, we found significant differences in survival based on p-STAT3 expression (Fig. 5C) (HR = 1.871; 95%CI: 0.993 - 3.525; n deaths = 95; *p* = 0.05).

## DISCUSSION

Our study is the first to demonstrate the negative prognostic influence of p-STAT3 expression in stage IV melanoma patients that did not develop CNS metastasis but not in those patients that did develop CNS metastasis. These findings are consistent with those of a previously published study examining the negative impact of p-STAT3 on the progression of human cutaneous melanoma [32]. Our results are also consistent with the previously demonstrated prognostic influence of p-STAT3 on survival in patients with other types of malignancies, such as glioma [27]. However, given the biological role of p-STAT3 in invasion, migration, and metastasis, we were surprised that p-STAT3 expression in systemic melanoma metastases was not associated with either the development of CNS metastasis or the time to first CNS metastasis. Furthermore, although we found differences in the p-STAT3 expression between different sites of metastasis, with the lung being the most common site [31], despite elevated p-STAT3 expression in the subset of patients with melanoma lung metastasis, these patients did not have a significantly higher risk of developing CNS metastasis.

Our secondary findings are in direct contrast with the results of a previous study, which demonstrated that p-STAT3 expression promotes CNS metastasis [14]. In that study, cloned human melanoma cell lines with enforced upregulation of constitutive STAT3 or blockade of STAT3 activity were evaluated for their ability to metastasize to the brain in murine models. In our study, the lack of an association between p-STAT3 expression and CNS metastasis may be attributed to the fact that the systemic tumor biology may not be reflective of the tumor microenvironment immediately preceding development of CNS metastasis. In fact, most of the systemic melanoma metastases that were resected and used in this analysis preceded the development of CNS metastasis by several years - ample opportunity for the biology to be altered. Another possible explanation for this discrepancy is the limitations of animal systems in recapitulating the biology of human melanoma patients. Ultimately, we suspect that the propensity to develop CNS metastasis is governed not by a single mechanism or pathway but rather by the combination and interaction of several key pathways and genes that ultimately influence outcome [33, 34].

Perhaps one of the more optimistic results of this study is the extended median survival time seen in stage IV melanoma patients, including those that developed CNS metastasis. In our analysis, the median survival time was 3.4 years in patients with stage IV melanoma that did not develop CNS metastasis (from the time of stage IV diagnosis), 1.9 years in those that developed CNS metastasis (from the time of stage IV diagnosis), and 1.0 years from the date of diagnosis of CNS metastasis; these compare favorably with historical data, which found a median survival time of less than 1 year in all patients with disseminated melanoma [35] and of only 3.8 months in patients with brain metastasis [8]. The current analysis was conducted with samples from patients treated at a single institution, which pursues aggressive surgical and multimodality treatment of metastatic melanoma, including in patients with brain metastasis that could bias these results in a favorable manner. Other recent series at institutions that pursue similar treatment schemas have demonstrated similar outcomes, both in the setting of melanoma patients with brain metastases [36] and in melanoma patients with limited systemic metastases amenable to complete surgical resection [37]. Despite the still grim prognosis of metastatic melanoma, these findings are certainly encouraging.

Our findings also inform the study design and stratification for planned clinical trials of p-STAT3 inhibitors. The data presented here indicate that stage IV melanoma patients that do not have CNS metastasis should be stratified on the presence or absence of p-STAT3 expression during efficacy clinical trials. One small molecule inhibitor of p-STAT3 with excellent CNS penetration, WP1066, has demonstrated antitumor effects in a murine model of melanoma, including against melanoma within CNS [38]. The *in vivo* effects of WP0166 are accompanied by a reduction of immunosuppression, including regulatory T cells (Tregs) and tumor-supportive M2 macrophages, with a concomitant increase in the cytotoxic immune response of T cells. These findings highlight the likely importance of immune cells on the effects of p-STAT3 inhibitors on tumor activity but also suggest that activity of p-STAT3 in the immune cell populations may play a significant role in influencing outcome. STAT3 has been shown to be a key transcriptional regulator of FoxP3 in Tregs [39], and Treg infiltration has been shown in melanoma patients to be a negative prognosticator [40] and a predictor of local recurrence [41]. Furthermore, M2 macrophages defined by their expression of p-STAT3 [42-44] have also been shown to support angiogenesis and growth in melanoma [45] and may also be a prognostic factor in melanoma patients [46]. Given that the tumor-infiltrating immune cells constitute a small minority of the overall tumor population, a limitation of the current study is that this influential subset may have not been fully taken into account in the context of the overall p-STAT3 expression.

In conclusion, as inhibitors of p-STAT3 move into clinical trials in patients with melanoma, including those with CNS metastasis, stratification by p-STAT3 should be considered for stage IV melanoma patients who have not developed CNS metastasis. Finally, given the current cumulative evidence, alterations in the clinical practice of surveillance brain magnetic resonance imaging need not be altered on the basis of p-STAT3 expression in systemic metastasis.
